# Failure rates and factors associated with infrazygomatic crestal orthodontic implants - A prospective study

**DOI:** 10.1016/j.jobcr.2023.02.010

**Published:** 2023-02-25

**Authors:** Gauri Gill, Keerthan Shashidhar, M.N. Kuttappa, Dhyan Kushalappa P B, Gautham Sivamurthy, Soham Mallick

**Affiliations:** a Gills Multispecialty Dental Solutions, Chandigarh, India; b Department of Orthodontics and Dentofacial Orthodontics, NITTE Deemed to Be University, AB Shetty Memorial Institute of Dental Sciences, Mangalore, Karnataka, India; c Department of Dentistry, Kodagu Institute of Medical Sciences, Madikeri, Karnataka, India; d School of Dentistry, University of Dundee, Ireland; e Dr Soham's Dental Clinic, Mumbai, Maharashtra, India

**Keywords:** Orthodontic anchorage procedures, Orthodontic appliance design, Bone screws, Inflammation

## Abstract

**Objective:**

Infrazygomatic crestal (IZC) implants have gained increased popularity over the past few years. Hardly any studies have been done to assess the rate and reasons for failure of IZCs. This prospective study was planned and designed with the primary objective of assessing the rate of failure of bone-screws (BS) placed in the infrazygomatic crest. In continuation, the secondary objective was to assess the factors that were associated with the failure.

**Materials and methods:**

The study was carried out by taking a detailed case history, (age, gender, vertical skeletal pattern, medical history), photographic records, radiographs, and clinical examination of a total of 32 randomly selected. patients of south indian origin who required infrazygomatic implants bilaterally as the choice of anchorage conservation to retract their incisors. All selected subjects were required to take a PA Cephalogram after the implant placement. The age of the patients ranged from 18 to 33 with an average age of 25 years. The patient log was maintained which included the treatment mechanics, status of oral hygiene, stability of implants, time of loading of the implant, presence of inflammation and time of failure of implant. The angulation of implant was measured on a digital PA cephalogram using Nemoceph software. These parameters were examined to evaluate independent and dependent variables using the Chi-Square test and Fischer's exact test.

**Result:**

A failure rate 28.1% for IZC placed in the infrazygomatic crest region was observed. Patients with a high mandibular plane angle, poor oral hygiene, immediately loaded implant, peri-implantitis, and severe clinical mobility showed higher failure rates. Variables such as age, gender, sagittal skeletal pattern, length of the implant, type of movement, occluso-gingival position, method of force application, and angle of placement were not significantly associated with implant failure.

**Conclusion:**

Oral hygiene and peri-screw inflammation must be controlled to minimize the failure of bone screws placed in the infrazygomatic crest region. Loading of the implant should be done after a latent period of two weeks. A higher failure rate was observed in patients with vertical growth pattern.

## Highlights

• Bone screws can be positioned in movable mucosa without risking failure.
• Loading bone screws after a period of two weeks had higher scope for success.
• Bone screw failure is highly influenced by poor oral hygiene & peri-implantitis.
• Individuals with vertical growth show an increased risk of failure of bone screws.
• Minimal bone screw mobility is acceptable if it can withstand orthodontic loading.

## Graphical abstract

**Figure:**
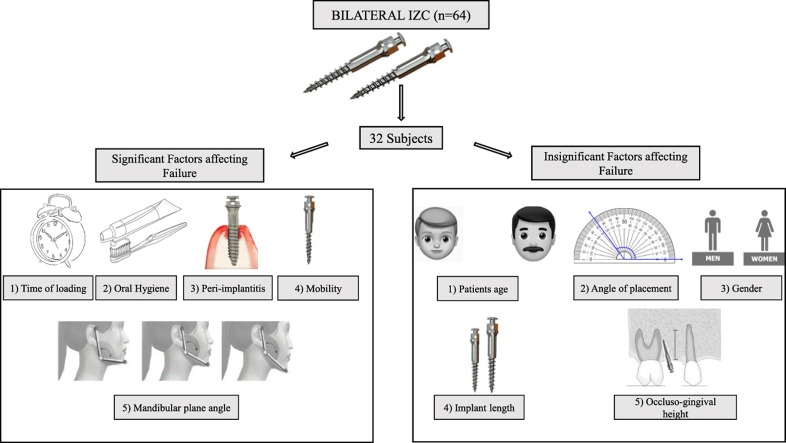

## 1 Introduction

Skeletal anchorage systems have revolutionized the way orthodontics is practiced, especially with mini-screws that are least invasive and clinician-friendly. Although traditionally mini-implants or mini-screws have been placed in the alveolar process and the palate, new sites, include the Infrazygomatic crest area (IZC), buccal shelf area, ramus, and nasal spine (referred to as extra-alveolars) have been proposed.^1^^,^^2^ Infrazygomatic crest of the maxilla is such an anatomical site away from the dentoalveolar region lying higher and lateral to the 1st and 2nd molar region. Thus allowing unhindered tooth movement, thereby reducing the probability of root contact.^3^ Due to a thick band of cortical bone, these sites provide for better primary stability.^3^

The mini implants used in these extra alveolar areas are relatively larger and commonly referred to as Bone Screws (BS).^4^ They have been successfully used as an absolute anchorage system in various tooth movements in sagittal and vertical dimensions, including open bite correction, simultaneous upper and lower arch distalization, and Intrusion of maxillary dentition.^5^^,^^6^

Osseointegration provides for stability in dental implants, lack of which clinically presents as mobility which is a sign of failure.^7^ On the other hand, retention of mini-implant screws is not by osseointegration but instead facilitated by mechanical interlocking at the implant-bone interface.^8^ Thus, the cortical bone thickness (CBT) is one of the vital aspects which determine the primary stability after the placement of micro-implants.^9^

For traditional mini-screws, minimal mobility might not be the cause for failure, as observed in a study by Park^7^ where 75% of minimally mobile screw implants were found to be successful.^7^

The literature evidence investigating specifically the rate of failure of mini-implants placed in the IZC is very sparse. The present study thus attempts to assess the failure rates of implants placed in the Infrazygomatic crest area and also investigates the factors associated with this outcome.

## 2 Materials and Methods

This prospective study was carried out in the Department of Orthodontics and Dentofacial Orthopedics, A B Shetty Memorial Institute of Dental Sciences, Mangalore, India, to assess the failure rates and factors associated with failure of Infrazygomatic Crest Implants. Clearance for the study was obtained from the Institutional Ethics Committee. (Cert No: ABSM/EC/62/2018).

The study was carried out by taking a detailed case history, (age, gender, vertical skeletal pattern, medical history), photographic records, radiographs, and clinical examination of a total of 32 randomly selected. patients of south indian origin who required infrazygomatic implants bilaterally as the choice of anchorage conservation to retract their incisors. All selected subjects were required to take a PA Cephalogram after the implant placement. The age of the patients ranged from 18 to 33 with an average age of 25 years. The patient log was maintained which included the treatment mechanics, status of oral hygiene, stability of implants, time of loading of the implant, presence of inflammation and time of failure of implant. The angulation of implant was measured on a digital PA cephalogram using Nemoceph software.

Patients with incomplete logs/records, previous history of orthodontic treatment, and/or orthognathic treatment, and/or facial trauma were excluded from the study. Patients who were medically compromised (Diagnosed Syndromes/Congenital defects/Facial Deformities) were also excluded from the study. The patient details, clinical features, status of the implant, and treatment progress were examined from the treatment log and photographic images to evaluate independent and dependent variables. Bone screws used in the clinical setup were 12/14 mm in length and 2 mm in diameter. The bone screws used in the study were from S K Surgicals. All implants were made from Titanium (Grade 5). The specification number for the implant material was ASTM B 265 Gr.5 : 2015. The composition of elements of the alloy used were 0.015% of Carbon, 0.28% of Iron, 0.015% of Nitrogen, 4.05% of Vanadium, 6.02% of Aluminium and 89.62% of Titanium. All implants were placed by a senior orthodontic faculty from the same college who had years of experience placing over 500 bone screws. Mini-implant failure was the primary outcome. Failure was defined as loss of the mini-screw in less than 8 months after placement. Independent variables associated with clinical success of the mini implant were age, gender, mandibular plane angle, length of the implant, side on which it failed, occluso-gingival position, angle of placement, time till loading, method of force application, oral hygiene, inflammation around the implant and implant mobility. These variables were examined as predictors of implant failure (Table 1).

**Table 1:** List of variables to be assessed.

	CHARACTERISTICS	
1	Age	<18 years
		≥18 years
2	Gender	Male
		Female
3	Length of the implant	12 mm
		14 mm
		All others
4	Side of implant failure	Left
		Right
5	Occluso-gingival Position	Upper attached gingiva
		Upper oral mucosa -low
		Upper oral mucosa -high
6	Angle of implant to the occlusal plane:	
	Right	0°- 45°
		46°-90
	Left	0°- 45°
		46°-90
7	Time till loading	Immediate
		After two weeks
8	Oral hygiene	Good
		Fair
		Poor
9	Inflammation	Yes
		No
10	Mobility	Yes
		No
11	Mandibular plane angle	High angle
		Average angle
		Low angle

### 2.1 Statistical analysis

The collected information was summarized by using frequency and percentage.

To identify factors associated with failure rate Chi-Square Test or Fischer's Exact Test was used. The p-value < 0.05 was considered significant.

## 3 Results

A total of 64 implants were placed, out of which 18 implants failed, and 46 remained stable, giving an overall failure rate of 28.1%. A failure rate of 31.3% was observed for the left side. A failure rate of 25.0% was observed for the right side (Table 2). Out of 32 patients who received the IZC mini-implants, 4 had unilateral failures, and 7 had bilateral failures (14 failed implants).

**Table 2:** Implant failure - Left side, Right Side and Overall.

		Frequency	Percentage
Left	Success	22	68.8
	Failure	10	31.3
Right	Success	24	75.0
	Failure	8	25.0
Overall	Success	46	71.9
	Failure	18	28.1

### 3.1 Comparison of implant failure according to age

A failure rate of 58.3% was observed in subjects below 18 years of age and a failure rate of 20% was observed in subjects aged 18 and above. On carrying out the Chi-Square test, the association was statistically insignificant (Table 3).

**Table 3:** Comparison of implant failure according to age.

Age	Overall Outcome		Total	Chi square test		Odds ratio (95% CI)
	Success	Failure		Chi square value	p-value	
< 18 years	14	10	24	3.48	0.06(NS)	0.35(0.11–1.08)
	58.3%	41.7%	100.0%			
> 18 years	32	8	40			
	80.0%	20.0%	100.0%			

*p < 0.05 Statistically Significant, p > 0.05 Non Significant, NS.

### 3.2 Comparison of implant failure according to time till loading of the implant

A failure rate of 39.1% was observed in implants that were loaded immediately. Contrasting to this, a success rate of 100% was observed in implants that were loaded adter two weeks. On carrying out the Chi-Square test, the association was highly significant (Table 4).

**Table 4:** Comparison of implant failure according to occluso-gingival position and time till loading.

		Overall Outcome		Total	Chi Square test		Odds ratio (95% CI)
		Success	Failure		Chi Square value	P-Value	
Occluso-gingival position	Upper attached gingiva	13	5	18	3.80	0.15(NS)	
		72.2%	27.8%	100.0%			
	Upper oral mucosa - Low	6	6	12			2.60(0.56–12.02)
		50.0%	50.0%	100.0%			
	Upper oral mucosa - High	27	7	34			0.67(0.18–2.54)
		79.4%	20.6%	100.0%			
Time till loading	Immediate	28	18	46	9.80	0.002*	0.61(0.48–0.77)
		60.9%	39.1%	100.0%			
	After Two Weeks	18	0	18			
		100.0%	0.0%	100.0%			

*p < 0.05 Statistically Significant, p > 0.05 Non Significant, NS.

### 3.3 Comparison of implant failure according to oral hygiene

A failure rate of only 20% was observed in subjects with good oral hygiene. A failure rate of 16.7% was observed in subejcts with fair oral hygiene. However, a failure rate of 55.6% was observed in subjects with poor oral hygiene. On carrying out the Chi-Square test, the association was statistically significant (Table 5). There are greater odds of association between poor oral hygiene and implant failure (Table 5).

**Table 5:** Comparison of implant failure according to oral hygiene, inflammation and mandibular plane angle.

		Overall Outcome		Total	Chi Square Test		Odds ratio (95% CI)
		Success	Failure		Chi Square Value	p-value	
Oral Hygiene	Good	8	2	10	9.37	0.009*	
		80.0%	20.0%	100.0%			
	Fair	30	6	36			0.80(0.14–4.75)
		83.3%	16.7%	100.0%			
	Poor	8	10	18			5.00(0.82–30.46)
		44.4%	55.6%	100.0%			
Inflammation	Absent	26	0	26	17.14	<0.001*	1.90(1.41–2.57)
		100.0%	0.0%	100.0%			
	Present	20	18	38			
		52.6%	47.4%	100.0%			
Mandibular Plane Angle	High angle	13	13	26	11.53	0.003*	
		50.0%	50.0%	100.0%			
	Average angle	23	5	28			0.15(0.05–0.51)
		82.1%	17.9%	100.0%			
	Low angle	10	0	10			
		100.0%	0.0%	100.0%			

*p < 0.05 Statistically Significant, p > 0.05 Non Significant, NS.

### 3.4 Comparison of implant failure according to inflammation

A failure rate if 47.4% was noted in subjects that had local inflammation around the implants. Whereas a success rate of 100% was observed in subjects that had no inflammtion around the implants. On carrying out the Chi-Square test, the association was highly significant (Table 5).

There are greater odds of association between the presence of local inflammation and implant failure (Table 5).

### 3.5 Comparison of implant failure according to the mandibular plane angle

Failure rates of 50%, 17.9% and 0% were observed in subjects with high mandibular plane angle, average mandibular plane angle and low manidbular plane angle respectively. Of the 10 implants placed in low angled patients, none failed, giving a success rate of 100.0%. On carrying out the Chi-Square test, the association was highly significant (Table 6).

**Table 6:** Comparison of implant failure according to mobility.

Mobility	Overall Outcome		Total	Chi Square Test		
	Success	Present		Chi Square value	p-value	
Absent	31	1	32	19.79	<0.001*	35.13(4.26–289.47
	96.9%	3.1%	100.0%			
Present	15	17	32			
	46.9%	53.1%	100.0%			

*p < 0.05 Statistically Significant, p > 0.05 Non Significant, NS.

### 3.6 Comparison of implant failure according to mobility

A failure rate of 53.1% was observed in subejcts who had mobile implants. Whereas a failure rate of 3.1% was observed in subejcts whose implants showed no signs of mobility. On carrying out the Chi-Square test, the association was highly significant (Table 6). There are greater odds of association between the presence of mobility and implant failure (Table 6).

### 3.7 Comparison of implant failure according to the angle of placement

A failure rate of 30% was observed in implants that were placed at an anglulation between 46° and 90°. On the contrary, a success rate of 100% was observed in implants that were placed at an angulation of 0°–45°. The association was statistically insignificant on carrying out the Fischer's Exact Test. (Table 7)**. Comparison of implant failure according to gender** (Table 8)**, the length of the implant** (Table 9), **and the occluso-gingival position** (Table 4) **were statistically not significant.**

**Table 7:** Comparison of implant failure according to their angle of placement.

Angle of placement	Overall Outcome		Total	Fisher's Exact Test	Odds Ratio (95% CI)
	Success	Failure		p-value	
0–45	4	0	4	0.57(NS)	1.43(1.21–1.69)
	100.0%	0.0%	100.0%		
45–90	42	18	60		
	70.0%	30.0%	100.0%		

*p < 0.05 Statistically Significant, p > 0.05 Non Significant, NS.

**Table 8:** Comparison of implant failure according to gender.

		Overall Outcome		Total	Fishers exact test	Odds ratio (95% CI)
		Success	Failure		p-value	
Gender	Male	7	5	12	0.29(NS)	0.47(0.13–1.73)
		58.3%	41.7%	100.0%		
	Female	39	13	52		
		75.0%	25.0%	100.0%		

*p < 0.05 Statistically Significant, p > 0.05 Non Significant, NS.

**Table 9:** Comparison of implant failure according to the length of the implant.

Length of implant	Overall Outcome			Chi Square test		Odds ratio (95% CI)
	Success	Failure		Chi Square value	p-value	
12 mm	21	11	32	1.24	0.27(NS)	0.54(0.18–1.62)
	65.6%	34.4%	100.0%			
14 mm	25	7	32			
	78.1%	21.9%	100.0%			

*p < 0.05 Statistically Significant, p > 0.05 Non Significant, NS.

## 4 Discussion

In the present study, 32 patients were evaluated, each of whom had infrazygomatic crest implants placed bilaterally for orthodontic anchorage. These implants were followed up for a minimum of 8 months, or until they fulfilled their intended purpose or failed. In our study, similar to Chris H. Chang,^11^ a temporary anchorage device (TAD) was considered to have failed if the mini-implant exfoliated or was deemed too mobile to act as an anchor.

A vast number of studies have predominantly focused on the rates of success of mini-implants placed at inter radicular sites, With success rates varying between 83.9% and 93.67%.^7^^,^^12^, ^13^, ^14^, ^15^

The variation in rates might be explained by varied types of mini-implants, different areas of placement, and varying management protocols. Therefore a direct comparison of the success rate might not be possible. However, by removing every likely cause of failure mentioned in each study, operators might be able to increase the chances of success.^7^

The findings of our study show a slightly lower success rate (71.9%) of the IZC BS (12–14 mm), Lower than that reported by Chris H. Chang^11^ - 93.7% for the 12 mm bone screw. The reason for the lower success rate in our study may be attributed to lower cortical bone mineral density in Indians.^16^

A higher failure rate was observed for implants placed on the left side (31.3%) than those placed on the right (25.0%). Similar results were observed by various authors. ^17^^,^^7^ This reflects on the technical sensitivity of the procedure and possibly other uncontrolled biological factors such as unilateral preference for mastication unequal level of oral hygiene among left and right-handed patients.

### 4.1 Comparison of implant failure according to age

Clinically we observed a higher failure rate (41.7%) in younger individuals than adults though the association was statistically insignificant. Similarly, two systematic reviews and meta-analyses^18^^,^^19^ conducted in this context concluded that a higher age group had a higher success rate than the lower age group. Studies have also shown that teenagers and young adults (below the age of 20) demonstrated a lower success rate (63.8%),^13^ (68.6%),^15^(80%)^20^ when compared to adults (above the age of 20) (83.5)^15^, (85–88).^20^

In contrast, a large number of retrospective studies and meta-analyses show no correlation between age and mini implant failure.^7^^,^^12^^,^^21^, ^22^, ^23^ The reason for such an outcome can be explained by the study of Lee et al.^24^^,^ who found that the patient's age influences mini-implant failure as younger patients have lower bone density and finer cortical bone. The relation between age and cortical bone thickness was also studied by Farnsworth et al.^25^ and Fayed et al.,^26^ both of who reported a statistically significant age-related difference in the buccal region of cortical bone-the bulk of the cortical bone was found to be increased in the adult group when compared with the adolescent group.

### 4.2 Comparison of implant failure according to gender

Statistically, no significant differences in the rate of failure of mini-implants between males and females were observed in our study, which is in concordance with many studies^7^^,^^21^^,^^24^^,^^27^, ^28^, ^29^, ^30^, ^31^, ^32^

### 4.3 Comparison of implant failure according to the length of the implant

In our study, an equal number of 12 mm and 14 mm long implants were placed, having a failure rate of 34.4% and 21.9%, respectively, but these differences were statistically insignificant. Despite the difference in lengths, the diameter was fixed at 2 mm.

The cortical bone thickness is about 1.1–1.3 mm, and the attached gingiva is 1.0 mm in the maxillary first molar region. IZC 7 site is usually the preferred site for TADs as the alveolar bone is thicker buccally to the second molars. The knowledge of the above was also considered in selecting the screw length, which was either 12 mm/14 mm in length and 2 mm in diameter, such that the screw threads would engage cortical bone securely and ensure primary stability.^33^

Few contrary studies have reported that length was a factor that influences stability, and success is directly related to it.^7^^,^^9^^,^^34^^,^^35^ Tseng et al.^34^ found an overall success rate of 91.1% for mini-screws and 100% for mini screws equal to or longer than 12 mm. They reported that the success rate increased with the length of the mini screw, but the difference was not statistically significant. A study by Murugesan on the Dravidian population concluded that the ideal IZC screw length for the Dravidian population was found to be 9–11 mm.^36^

### 4.4 Comparison of implant failure according to time till the loading

On comparing implant failure with immediate and delayed time till loading, the results obtained in our study show a 100% success rate in delayed loaded implants group – loaded after 2 weeks compared to those loaded immediately (60.9%). The association was highly significant. We observed that loading the implant after 2 weeks decreases the risk of failure, as justified by an odd's ratio of 0.61.

In concordance with our study, three other studies considered this and indicated that deferred loading presented lower failure rates, though the finding was statistically non-significant.^37^, ^38^, ^39^

Cheng et al.^12^ reported a success rate of 89% on applying orthodontic forces after 2–4 weeks. While Costa et al.^40^ found almost the same results with immediate loading (87.5%).

Similarly, studies by Kuroda et al.^31^ and Manni A^41^ have reported advantages of immediate loading, whereas studies by Chen YJ et al.^42^ and Chung KR^43^ reported the disadvantages.

Differences may be attributed to the type of bone the implant was placed in, with dense mature bone responding better to immediate loading. In most cases, premature loading leads to healing by forming fibrous tissue between the bone and the mini-implant. ^10^Motoyoshi et al.^39^ also reported that delaying loading for 3 months is recommended to improve the success rate of orthodontic mini-implants placed in alveolar bones in adolescent patients. In our study, a 2-to-3 week latent period was used.

In a metanalysis by Papageorgiou,^10^ they concluded no significant differences in the failure rates of mini-screw implants were observed concerning the time of orthodontic force application: i.e., immediate loading (up to 2 weeks) or late loading (later than 2 weeks) (3 studies). Similarly, Nkenke et al.^44^ found no significant difference in daily bone apposition, bone-implant contact, and bone density in the presence or absence of early loading.

The results of a study by Park^7^ suggested that if the applied force is lower than 2 N, immediate loading of an anchor implant is possible. They recommended immediate loading to the implant anchor. Such immediate loading is probably possible because of successful mechanical interdigitation between the implant anchor and the alveolar bone.

### 4.5 Comparison of implant failure according to oral hygiene

The present study observed a higher failure rate in patients with poor oral hygiene (55.6%) than patients with good and fair oral hygiene. Oral hygiene is known to be a local risk factor for mini-implant failure since the stability of the mini implant depends on adequate oral hygiene.^20^^,^^45^

There is a strong statistical correlation between oral hygiene and failure rates in our study, and poor oral hygiene is associated with greater odds of failure. Although an expected finding, the contribution of oral hygiene in mini-screw failure is quite controversial. Sharma et al.^46^ reported that mini-implant failure was associated with poor oral hygiene and local inflammation, which was in concordance with our study. On the other hand, Park et al.^7^ stated that oral hygiene played no role, but local inflammation around the mini-implant does.

Kuroda et al.^31^ observed it was hard to maintain good oral hygiene in the posterior maxilla making the TAD more susceptible to peri-implant inflammation.

According to Chris Chang,^47^ a minimum of 5 mm of clearance from the head of the bone screw to the soft tissue should be maintained for efficient oral hygiene maintenance and to facilitate control of peri-screw inflammation. Inflammation damages the bone around the neck of the bone screws; progressive damage to the cortical bone causes mobility and exfoliation of the implant.^45^

### 4.6 Comparison of implant failure according to inflammation

Our study recorded that bone screws with signs of local inflammation (redness or swelling) showed a significantly lesser success rate (52.6%) than the 100% success rate of bone screws that presented with no signs of local inflammation around them. The odds ratio of 1.90 showed an increased risk of failure in implants with local inflammation.

C.H.Moon^48^ reported that inflammation damages bone surrounding the MI, and progressive damage to cortical bone endangers the MI. Our results are similar to previously done studies^7^^,^^10^^,^^20^^,^^42^ which also showed peri-implant inflammation as one of the most important factors to predict implant failure.

Cheng S-J et al.^12^ and Miyawaki et al.^20^ also concluded that implant placement on non-keratinized movable mucosa could cause local inflammation. On the contrary, Eric Hsu^47^ found no significant difference in the failure rates of IZC screws placed in the attached gingiva (6.23%) and movable mucosa (6.48%).

To prevent mobility of the mini-screw, prevention of local inflammation is of utmost importance.^20^ The risk for local inflammation around the mini-screw can be reduced by using appropriate oral hygiene measures.^13^

### 4.7 Comparison of implant failure according to occluso-gingival position

The association between the failure rate for implants placed in attached or movable mucosa was statistically insignificant.

### 4.8 Comparison of implant failure according to the mandibular plane angle

In this study, a 100% success rate was observed for patients with a low mandibular plane angle (horizontal growth pattern) followed by average angle patients (82.1%) and a far lower success rate of 50% for high-angle patients (vertical growth pattern). A highly significant association was found between the mandibular plane angle and the failure rate of the bone screw. This could be since retention of the BS depends upon mechanical interlocking at the implant-bone interface.^8^ Thus, bone density and cortical bone thickness become one of the most vital aspects that prevent premature loosening of the TAD, determining the primary stability after placement of mini-implants.^9^^,^^20^^,^^49^^,^^50^ A greater cortical bone thickness was also related to a decreased deflection of the mini implant.^51^ When mini-screws are inserted into a cortical bone thinner than 1 mm, skeletal anchorage is not ensured.^52^

However, some authors did not find any beneficial effects of increased overall bone density at the insertion site.^9^ Kuroda et al.^31^ argued that there wasn't any correlation between the rates of success of mini-screws and MPA.

The thickness of cortical bone varies with varying mandibular plane angles. The buccal cortical bone in subjects with a higher mandibular plane angle was thinner compared to subjects with a low angle. ^53^Our results are in concordance with other authors,^20^^,^^52^^,^^54^^,^^55^ all of whom suggest that there was an increased stability of mini-implants in patients with thicker cortical bone, and the cortical bone was thicker in people having low angle facial pattern than the group possessing high angle facial pattern. Swasty et al.^56^ also stated that the subjects with a vertical growth pattern had a decreased CBT at almost all sites. Horner et al.^54^ reported that the cortical bone was 0.08–0.64 mm thicker in the hypodivergent than in the hyperdivergent subjects.

A recent study by P. Paul et al.^57^ reported that cortical bone thickness in the infrazygomatic region was related to various facial types. It was significantly higher for subjects with a low mandibular plane angle between first and second molars as well as on the distal aspect of the second molars. This could be the reason why there was a 100% success rate in low-angle patients. However, this directly contradicts another recent study by Vargas,^58^ which reported no correlation between the subjects’ vertical face height and the cortical bone width in the Infrazygomatic Crest region.

### 4.9 Comparison of implant failure according to mobility

On comparing implant failure with the presence or absence of mobility, we observed that in the absence of mobility, there was a 96.9% success rate. In contrast, the success rate fell to 46.9% if the bone screw showed mobility. Mobility was found to be a highly significant factor in our study. There is an increased risk for failure of bone screws showing signs of mobility (odds ratio 35.13). Unlike dental implants, where mobility represents a lack of osseointegration leading to failure, mobility in screw implants for orthodontic anchorage mobility might not represent failure.^7^ This is also seen in our study; out of 32 implants showing mobility, 15 were stable enough to act as a stationary anchor and withstand orthodontic loading. Mobility of the implants was followed up every month from initial stability after placement to a minimum period of 8 months or till it was no longer required. This is consistent with the findings of Liou et al.,^59^ who specifically evaluated Infrazygomatic crest mini-implants and found that these screws have some degree of mobility without causing failure.

Dental implants are loaded with forces in all directions, but orthodontic screw implants are loaded with unidirectional lateral forces. Therefore minimal mobility can be allowed in screw implants.^7^ On the other hand, severe clinical mobility was a risk factor for implant failure.

A significant limitation in assessing mobility is that the grade of mobility is very subjective as there is no standardized grading to assess the mobility of mini-implants. Park et al.^7^ reported a higher failure rate for mini implants showing clinical mobility (24.4%) as compared with those that were clinically stable (1.4%)

Initial Stability is influenced by the quality and quantity of cortical bone.^28^ Data from animal studies indicate that mini-implant mobility seems to be negatively associated with insertion torque and positively correlated with bone mineral density and thickness of the cortical bone. Still, a study in rats showed that a significant decrease in mobility after 3 weeks was linked to a good prognosis for subsequent stability.^60^Miyawaki et al.^20^ stated that the prevention of inflammation around the peri-screw tissue was of utmost importance in preventing mobility.^20^

### 4.10 Comparison of implant failure according to their angle of placement

On comparing implant failure to the angle of placement, we found that implants placed at a 0 °- 45° angulation had a success rate of 100%, whereas those placed at an angulation of 46°–90° degree had a lower success rate of 70.0%. Though clinically relevant, the association was statistically insignificant.

While this study successfully determined the failure rates and failure factors of IZC screws, it did have certain limitations. One of the limitations was the type of implants sourced. The implants used in this study were sourced from a local manufacturer. This could have contributed to the overall failure rates. Another limitation was the assessment of the mobility of implants. This is a limitation because the grade of mobility is very subjective since there is no standardized grading to assess the mobility of mini-implants.

## 5 Conclusion

The following conclusions can be deduced:

- Bone screws placed in the Infrazygomatic crest region had a 28.1% failure rate.
- Bone screws placed on the left side had a higher failure rate (31.3%) than those on the right side (25.0%).
- Screws can be positioned in movable mucosa without risking failure, provided there was adequate soft tissue clearance (approximately 5 mm).
- Late loaded implants i.e., loading after a latent period of two weeks, had higher scope for success than those loaded immediately.
- Bone screw failure is highly influenced by poor oral hygiene and peri-screw inflammation.
- Bone screws in Individuals with a high mandibular plane angle have an increased risk of failure due to a thinner cortical bone
- Not all mobile screws fail clinically; minimal mobility is acceptable if they withstand orthodontic loading.
- Factors such as age, gender, length of the implant (12mm/14 mm), occluso-gingival position, method of force application, and placement angle were not significantly associated with higher or lower odds of mini-implant failure.

Bone screws have an excellent prognosis. Proper case selection and following the recommended protocol are essential to minimize failures. Excluding all of the above-highlighted causes might increase the chances of clinical success of implant mechanics.
